# Supplementary material for: CRISPR/Cas9-Based Mutagenesis of Histone H3.1 in Spinal Dynorphinergic Neurons Attenuates Thermal Sensitivity in Mice
Source: Int J Mol Sci. 2022 Mar 15;23(6):3178. doi: 10.3390/ijms23063178 (PMC8955318; doi:10.3390/ijms23063178)
Supplement: Supplementary file 1 [file ijms-23-03178-s001.zip › ijms-1541263-supplementary.pdf]

CRISPR/Cas9-Based Mutagenesis of Histone H3.1 in Spinal Dynorphinergic Neurons Attenuates Thermal Sensitivity in Mice

Mészár et al.

Supplementary Figure S1

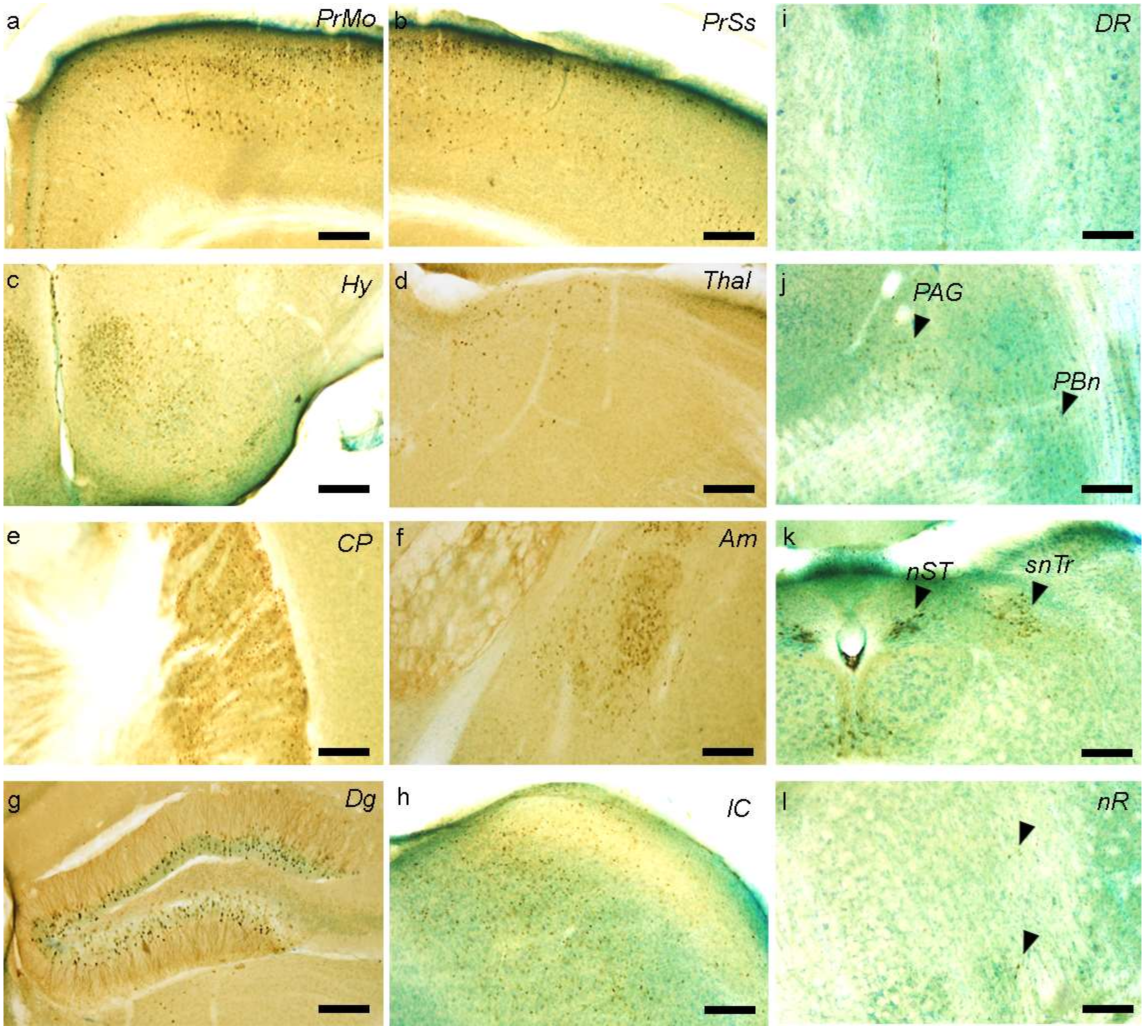

Conventional immunohistochemistry against EGFP on coronal sections of the whole brain of a Pdyn::cas9-EGFP mouse

Representative images of the horseradish peroxidase-DiAminoBenzidine conversion of the EGFP signal of Pdyn neurons in coronal sections from a Pdyn::cas9-EGFP hybrid mouse, at the level of the telencephalon/diencephalon, (**a-g**), midbrain (**h-j**) and the medulla (**k**,**l**). The sections have been counterstained with Toluidine blue to enhance the contrast of the brown cell bodies. *PrMo*, primary motor area; *PrSs*, primary somatosensory area layer 2/3 and layer 5; *Dg*, dentate gyrus; *Thal*, mediodorsal nucleus of the thalamus; *Hy*, the dorsomedial nucleus of the

hypothalamus; *CP*, caudoputamen; *Am*, central amygdalar nucleus; *IC*, inferior colliculus; *PAG*, periaqueductal gray; *PBn*, parabrachial nucleus; *DR*, dorsal nucleus raphe; *nST*, the nucleus of the solitary tract; *snTr*, the spinal nucleus of the trigeminal nerve; *nR*, reticular nucleus. Scale bars are 200 μm.

Supplementary Figure S2

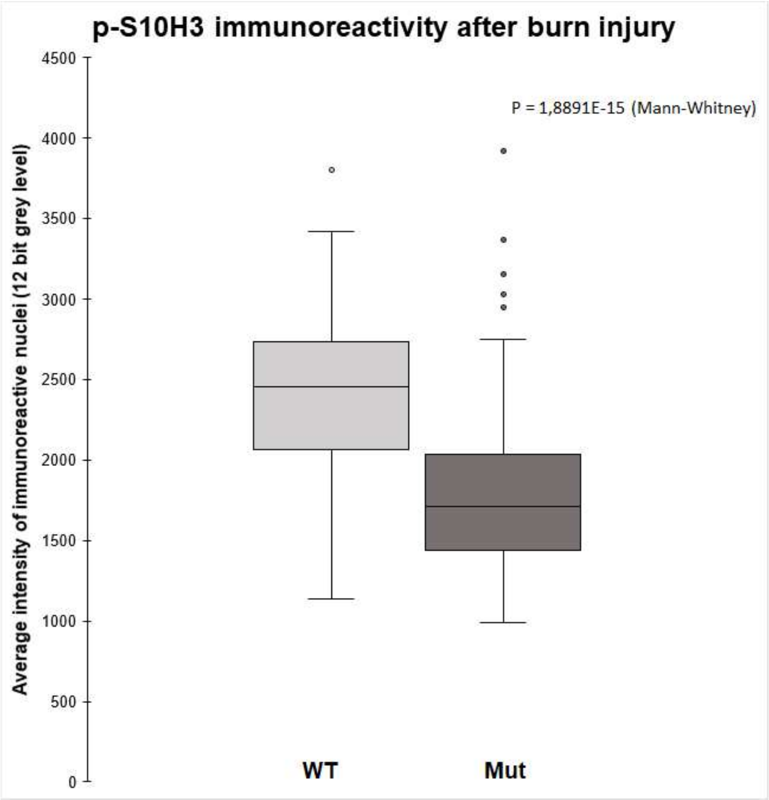

Evaluation of p-S10H3 immunoreactivity after burn injury

Burn injury-induced p-S10H3 immunoreactivity (IR) was reduced after transduction with AAV9_mutH3.1 as assessed by intensity profile analysis of p-S10H3-IR nuclei. The blox plot indicates the average grey level values of p-S10H3-IR nuclei in spinal cord samples from transduced (AAV9_mutH3.1; Mut) and non-transduced animals (WT). Y-axis represents the average grey level intensity of p-S10H3-IR nuclei. Dots are outlier values are also indicated in the box plot.

Supplementary Figure S3

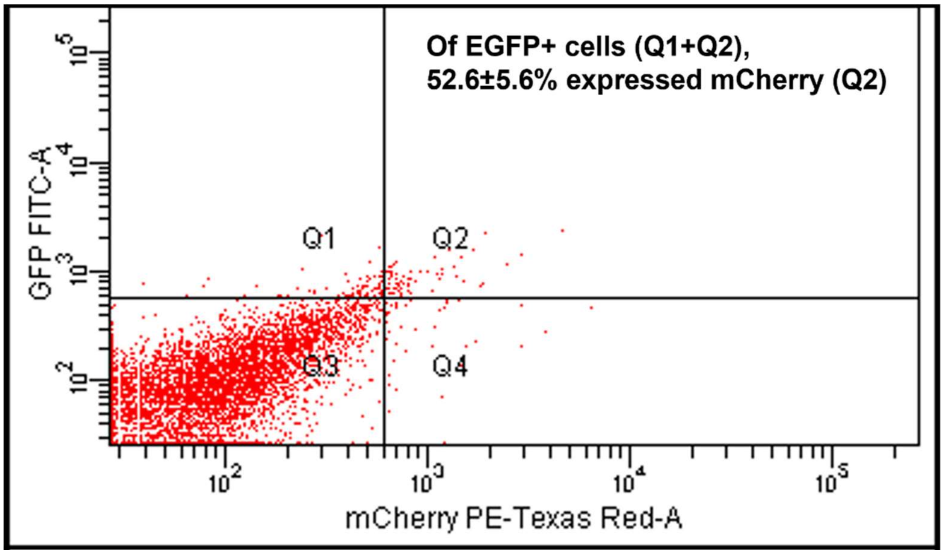

Population	#Events	%Parent
☒ Q1	63	0.9
☒ Q2	56	0.8
☒ Q3	6,611	97.7
☒ Q4	38	0.6

FACS gating strategy to quantify double labelled (EGFP+/mCherry+) dynorphinergic neurons in Pdyn::cas9-EGFP mice

Three days after post-infection with AAV9_mutH3.1, fluorescence-activated cell sorting (FACS) was performed to determine the percentage of EGFP-positive Pdyn neurons in the spinal cord that express mCherry (*i.e.* double tagged neurons).

Scatter plots of green fluorescence intensity on the x-axis (arbitrary units; excitation 488 nm/emission 510/20 nm) and red fluorescence intensity on the y-axis (arbitrary units; excitation 586 nm/emission 610/20 nm) from a representative FACS experiment demonstrates that about half of EGFP-expressing Pdyn neurons were infected by the AAV9_mutH3.1 based on their mCherry fluorescence.

Supplementary Figure S4

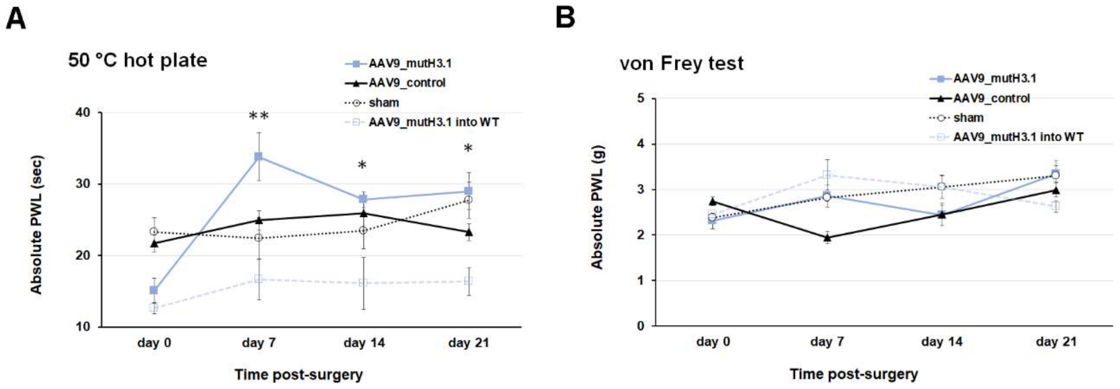

Absolute values of paw withdrawal latency to thermal- and mechanical pain

Changes in paw withdrawal latencies (PWL) to thermal- (A) and mechanical (B) stimuli were evaluated before (day 0) and after the surgery (day7, 14, 21) in different groups of Pdyn::cas9-EGFP mice (*i.e*. AAV9_mutH3.1, AAV9_control, sham-operated) and also in wild-type animals transduced with AAV9_mutH3.1. Values at Day 0 represent the pre-surgery baseline values (BTM). (A) *$p < 0.05$ and **$p < 0.01$ compared with the BTM ($p = 0.007$ on day 7, $p = 0.01$ on day 14 and 21, *non*-parametric Mann-Whitney U test). (B) Absolute values of paw withdrawal latency to painful mechanical stimuli showed no significant alterations within, and differences between the groups.

Supplementary Figure S5

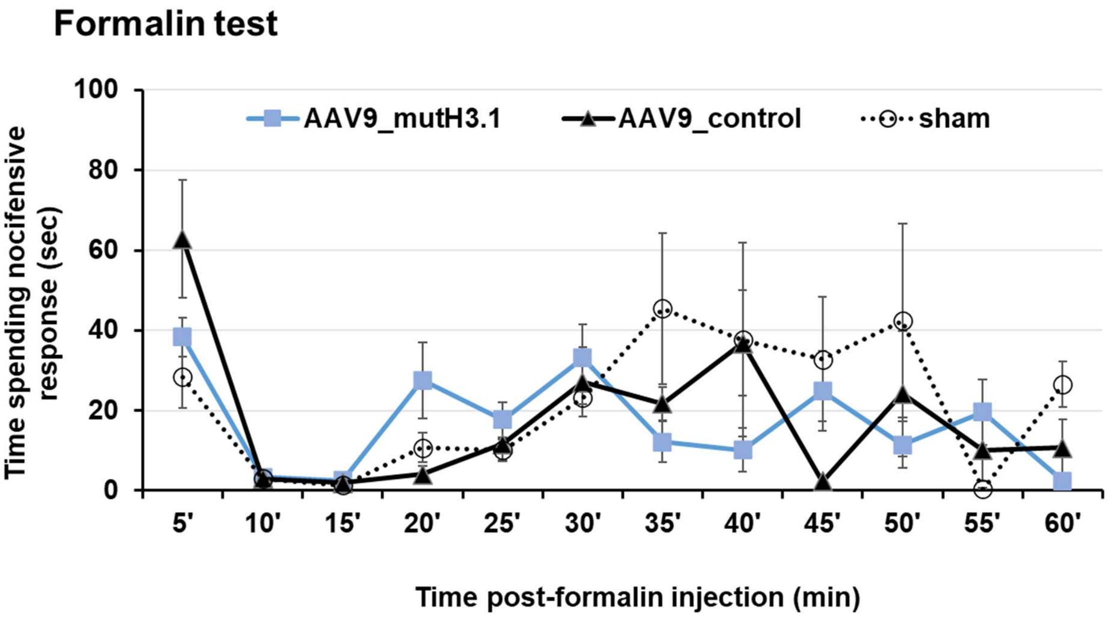

Assessment of formalin-induced somatic pain

This behavior was quantified as the length of time spent performing a nocifensive response (licking, biting, and shaking) in 5 min periods across an hour after application of formalin. Raw data of the time spent with formalin-induced pain-related movements in each group are presented. There were no statistically significant differences between the groups, at 0.05 level. See also **Supplementary Table S4** for statistical information.

Supplementary Table S1. Key Resources

REAGENT	COMPANY	CATALOG OR STOCK NUMBER / version information / external link
**Experimental animals**		
Pdyn-IRES-Cre mice	The Jackson Lab. ME, USA	# 027958
Rosa26-LSL-Cas9	The Jackson Lab. ME, USA	# 026175
C57BL/6	AnimaLab Hungary Kft, Hungary	https://animalab.eu/
**Viral preps**		
Custom made-AAV9_mutH3.1.1	SignaGen Labs. MD, USA	# SL100865
pAAV-EF1a-double floxed-hChR2(H134R)-mCherry-WPRE-HGHpA	addgene, MA, USA	Addgene viral prep # 20297-AAV9
**Software/device**		
sgRNAs designing	broadinstitite.org	https://portals.broadinstitute.org/gpp/public/analysis-tools
hot/cold plate	Ugo Basile, Italy	#35150
dynamic plantar aesthesiometer	Ugo Basile, Italy	#161
Snapgene 5.1.0	GSL Biotech LLC, CA, USA	https://www.snapgene.com
Olympus FV3000 confocal systems	Olympus, Tokyo, Japan	https://www.olympus-global.com/
Neurolucida	MBF BioScience, Williston, Vermont, USA	v11.07
FACS Aria III	BD Biosciences, CA, USA	https://www.bdbiosciences.com/
FACSDiva 6.1.3	BD Biosciences, CA, USA	https://www.bdbiosciences.com/
Vibrating blade vibratome	Leica Biosystems, Wetzlar, Germany	VT 1000S
FV31S-DT	Olympus, Tokyo, Japan	https://www.olympus-global.com/
Scyscan1272 X-ray High-Resolution Microtomograpy (micro-CT)	Bruker,Kontich, Belgium	https://www.bruker.com/en/products-and-solutions/preclinical-imaging/micro-ct/skyscan-1272.html
SkyScan NRecon software (version 2.0.4.2)	Micro Photonics Inc, PA, USA	https://www.microphotonics.com/products/micro-ct/nrecon-reconstruction-software/
RadiAnt DICOM Viewer & 3D Volume rendering tool	Medixant, Poznań, Poland	https://www.radiantviewer.com/

OriginPro9	OriginLab Corp., MA, USA	https://www.originlab.com/
Primer3Plus		https://primer3plus.com/
Fiji		https://imagej.net/software/fiji/
**Chemicals and tools**		
Alzet osmotic pump	DURECT Corporation, CA, USA	#1003D
5-0 coated vicryl absorbable suture	Ethicon NJ, USA	#J391H
DAB Peroxidase substrate kit	Vector Labs. Inc., CA, USA	#SK-4100
ExtAvidin peroxidase conjugate	Sigma, MI, USA	#E-2886
TRIzol™ Reagent	Thermo Fisher Sci. Inc. MA, USA	#15596026
High-Capacity cDNA Reverse Transcription Kit	Thermo Fisher Sci. Inc. MA, USA	#4368814
RNasin® Ribonuclease Inhibitor	Promega, WI, USA	# N2111
DreamTaq DNA Polymerase	Thermo Fisher Sci. Inc. MA, USA	#EP0701
BenchTop 100 bp DNA ladder	Promega	#G8291
**Primary antibodies**		
Chicken polyclonal to GFP	abcam, Cambridge, UK	#ab13970
Rat monoclonal [5F8] to Red Fluorescent Proteins (RFP)	Chromotek GmbH, Planegg-Martinsried, Germany	#5f8-100
p-S10H3	abcam Cambridge, UK	#ab5176
Guinea pig Polyclonal ProDynorphin antibody	GeneTex Irvine, CA, USA	# GTX10280
**Secondary antibodies**		
Goat anti-Rat IgG, Alexa Fluor 555	Thermo Fisher Sci. Inc. MA, USA	#A-21434
Alexa Fluor® 488 AffiniPure Donkey Anti-Chicken IgY	Jackson ImmunoResearch Europe Ltd, Cambridgeshire, UK	#703-545-155
Alexa Fluor® 647 AffiniPure Donkey Anti-Guinea Pig IgG	Jackson ImmunoResearch	#706-605-148

	Europe Ltd, Cambridgeshire, UK	
Biotin-SP-AffiniPure Donkey Anti-Chicken IgY	Jackson ImmunoResearch Europe Ltd, Cambridgeshire, UK	#703-065-155
Neurobasal medium	Thermo Fisher Sci. Inc., MA, USA	#21103049
Glutamax supplement	Sigma, MI, USA	#135050-061
7.5% Na-bicarbonate	Thermo Fisher Sci. Inc., MA, USA	#25080
papain	Merck Life Sci., NJ, USA	#107144
hyaluronidase type I	Merck Life Sci., NJ, USA	#H-3506
Trypsin-EDTA (0.25%), phenol red	Thermo Fisher Sci. Inc., MA, USA	#25200-056
FBS	Sigma, MI, USA	#F9665
Nylon net filter	Millipore, MA, USA	# NY4100010

Supplementary Table S2

Primer sequences applied in this study

Name	5'-3' sequence
STOP-cas9 WT_rev[#]	CAG GAC AAC GCC CAC ACA
STOP-cas9 mut_for[#]	TCC CCA TCA AGC TGA TCC
STOP-cas9 mut_rev[#]	CTT CTT CTT TGG GGC CAT CT
STOP-cas9 WT_for[#]	AAG GGA GCT GCA GTG GAG TA
cre_for[$]	GCA TTA CCG GTC GAT GCA ACG AGT GAT GAG
cre_rev[$]	GAG TGA ACG AAC CTG GTC GAA ATC AGT GCG
mCherry_for*	GGGCGAGGAGGATAACATGG
mCherry_rev*	GATGTTGACGTTGTAGGCGC
GAPDH_for*	ACCCATCACCATCTTCCA
GAPDH_rev*	CATCACGCCACAGCTTTCC

[#]Primer set for genotyping Rosa26-LSL-Cas9 line was recommended by The Jackson Laboratory.

[$]Primer set for genotyping Pdyn^{cre} line was recommended by the Mouse Genetics Core (https://mgc.wustl.edu/).

*Primer sets were designed by using Primer3Plus.

Supplementary Table S3.

sgRNAs sequences targeting wild-type histone H3.1

Name	Orientation	PAM sequence	sgRNA sequence
sgRNA1	antisense	AGG	TGTCCTCAAACAGACCCACA
sgRNA2	antisense	CGG	GCCGCCGGTGGACTTGCGAG
sgRNA3	antisense	GGG	GGTGGCTAGCTGCTTGCGCG

The input sequence was the Mus musculus H3 clustered histone 1 (H3c1; Gene ID: 360198). This gene is intronless and encodes a replication-dependent histone that is a member of the histone H3 family. sgRNAs sequences targeting H3 clustered histone 1 (*i.e*. histone H3.1) was designed with the aid of broadinstitute.org (see **Supplementary Table S1**).

Supplementary Table S4. Statistical analysis

Figure	Comparison	Analysis	p/U/χ²/df values	number of animals
Fig4b	The overall influence of the treatment with the AAV9_mutH3.1 on paw withdrawal latency (PWL) in response to noxious heat	Kruskal-Wallis ANOVA	p=0.009<br>df=3<br>χ^2=11.533	7
Fig4b	The overall influence of the treatment with the AAV9_control on PWL in response to noxious heat	Kruskal-Wallis ANOVA	p=0.487<br>df=3<br>χ^2=2.433	6
Fig4b	The overall influence of the sham operation on PWL in response to noxious heat	Kruskal-Wallis ANOVA	p=0.460<br>df=3<br>χ^2=2.580	4
Fig4b	PWL in response to noxious heat of AAV9_mutH3.1-treated *vs* sham between the corresponding days	Non-parametric Mann-Whitney test	day7: p=0.006<br>U=27;<br>day14: p=0.012<br>U=27;<br>day21: p=0.029<br>U=26	7 AAV9_mutH3.1-treated and 4 sham treated
Fig4b	PWL in response to noxious heat of AAV9_mutH3.1-treated *vs* AAV9_control between the corresponding days	Non-parametric Mann-Whitney test	day7: p=0.001<br>U=42;<br>day14: p=0.013<br>U=38;<br>day21: p=0.001<br>U=42	7 AAV9_mutH3.1-treated and 6 AAV9_control treated
Fig4c	The overall influence of the treatment with the AAV9_mutH3.1 on PWL in response to mechanical force	Kruskal-Wallis ANOVA	p=0.106<br>df=3<br>χ^2=6.110	4

Fig4c	The overall influence of the treatment with the control-AAV9 on PWL in response to mechanical force	Kruskal-Wallis ANOVA	p=0.106<br>df=3<br>χ^2=6.110	3
Fig4c	The overall influence of the sham operation on PWL in response to mechanical force	Kruskal-Wallis ANOVA	p=0.075<br>df=3<br>χ^2=6.897	3
Fig4c	PWL in response to mechanical pain of AAV9_mutH3.1-treated *vs* sham between the corresponding days	Non-parametric Mann-Whitney test	day7: p=0.4<br>U=9;<br>day14: p=0.4<br>U=3;<br>day21: p=0.859<br>U=7:	4 AAV9_mutH3.1-treated and 3 sham treated
Fig4c	PWL in response to mechanical pain of AAV9_mutH3.1-treated *vs* AAV9_control treated mice between the corresponding days	Non-parametric Mann-Whitney test	day7: p=0.228<br>U=10;<br>day14: p=0.857<br>U=7;<br>day21: p=0.114<br>U=11	4 AAV9_mutH3.1-treated and 3 AAV9_control treated mice
Fig4d	The overall influence of the treatment with the AAV9_mutH3.1 on the 1st phase of the formalin response	Kruskal-Wallis ANOVA	In the 1st phase:<br>p=0.290<br>df=2<br>χ^2=2.474	6 AAV9_mutH3.1<br>3 AAV9_control<br>and 3 sham
Fig4d	The overall influence of the treatment with the AAV9_mutH3.1 on the 2nd phase of the formalin response	Kruskal-Wallis ANOVA	In the 2nd phase:<br>p=0.148<br>df=2<br>χ^2=3.807	6 AAV9_mutH3.1<br>3 AAV9_control<br>and 3 sham
Fig4d	Integrated time in response to formalin in the groups of AAV9_mutH3.1-	Non-parametric Mann-Whitney test	In the 1st phase:<br>p=1<br>U=9<br><br>In the 2nd phase:	6 AAV9_mutH3.1 treated and<br>3 AAV9_control treated mice

	treated *vs* AAV9_control treated mice		p=1<br>U=9	
Fig4d	Integrated time in response to formalin in the groups of AAV9_mutH3.1-treated *vs* sham operation	Non-parametric Mann-Whitney test	In the 1st phase:<br>p=0.166<br>U=3<br><br>In the 2nd phase:<br>p=0.166<br>U=3	6 AAV9_mutH3.1 treated and 3 sham-operated mice
Fig4d	Integrated time in response to formalin in the groups of control_AAV9-treated *vs* sham operation	Non-parametric Mann-Whitney test	In the 1st phase:<br>p=0.4<br>U=2<br><br>In the 2nd phase:<br>p=0.1<br>U=0	3 AAV9_control treated and 3 sham-operated mice
Fig4e	The overall influence of the treatment with the AAV9_mutH3.1 on body weight	Kruskal-Wallis ANOVA	p=0.32<br>df=3<br>χ^2=3.472	7
Fig4e	The overall influence of the treatment with the control-AAV9 on body weight	Kruskal-Wallis ANOVA	p=0.141<br>df=3<br>χ^2=5.453	5
Fig4e	The overall influence of sham operation on body weight -	Kruskal-Wallis ANOVA	p=0.975<br>df=3<br>χ^2=215	4
Fig4e	Changes in body weight of AAV9_mutH3.1-treated *vs* sham operation between the corresponding days	Non-parametric Mann-Whitney test	day7: p=0.109<br>U=5;<br>day14: p=0.927<br>U=13;<br>day21: p=0.763<br>U=12	7 AAV9_mutH3.1-treated and 4 sham operated mice
Fig4e	Changes in body weight of AAV9_mutH3.1-treated *vs* AAV9_control treated mice between the corresponding days	Non-parametric Mann-Whitney test	day7: p=0.267<br>U=10;<br>day14: p=0.755<br>U=15;<br>day21: p=0.972<br>U=18	7 AAV9_mutH3.1-treated and 5 AAV9_control treated mice

Supplementary Data S1.

The complete sequence of the designed construct with color codes corresponds to **Fig. 2c** in the main text. Bases in black represent segments without any annotated function.

ccatagagcccaccgcatccccagcatgcctgctattgtcttcccaatcctcccccttgctgtcctgccccaccccaccccccagaatagaatgacacctactcag
acaatgcgatgcaatttcctcattttattaggaaaggacagtgggagtggcaccttccagggtcaaggaaggcacgggggaggggcaaacaacagatggctgg
caactagaaggcacagtcgaggctgatcagcgggtttaaacgggccctctagactcgagttacttgtacagctcgtccatgccgccggtggagtggcggccctcg
gcgcgttcgtactgttccacgatggtgtagtcctcgttgtgggaggtgatgtccaacttgatgttgacgttgtaggcgccgggcagctgcacgggcttcttggcctt
gtaggtggtcttgacctcagcgtcgtagtggccgccgtccttcagcttcagcctctgcttgatctcgcccttcagggcgccgtcctcggggtacatccgctcggagg
aggcctcccagcccatggtcttcttctgcattacggggccgtcggaggggaagttggtgccgcgcagcttcaccttgtagatgaactcgccgtcctgcagggagg
agtcctgggtcacggtcaccacgccgccgtcctcgaagttcatcacgcgctcccacttgaagccctcggggaaggacagcttcaagtagtcggggatgtcggcg
gggtgcttcacgtaggccttggagccgtacatgaactgaggggacaggatgtcccaggcgaagggcagggggccaccettggtcaccttcagcttggcggtctg
ggtgccctcgtaggggcggccctcgccctcgccctcgatctcgaactcgtggccgttcacggagccctccatgtgcaccttgaagcgcatgaactccttgatgatg
gccatgttatcctcctcgcccttgctcaccatggtggcgcagtgggttctctagttagccagagagctctgcttatatagacctcccaccgtacacgcctaccgccc
atttgcgtcaatggggcggagttgttacgacattttggaaagtcccgttgattttggtgccaaaacaaactcccattgacgtcaatggggtggagacttggaaatc
cccgtgagtcaaaccgctatccacgcccattgatgtactgccaaaaccgcatcaccatggtaatagcgatgactaatacgtagatgtactgccaagtaggaaag
tcccataaggtcatgtactgggcataatgccaggcgggccatttaccgtcattgacgtcaatagggggcgtacttggcatatgatacacttgatgtactgccaagt
gggcagtttaccgtaaatactccacccattgacgtcaatggaaagtccctattggcgttactatgggaacatacgtcattattgacgtcaatgggcgggggtcgtt
gggcggtcagccaggcgggccatttaccgtaagttatgtaacgcggaactccatatatgggctatgaactaatgaccccgtaattgattactattaataactagtc
aataatcaatgtcaacgcgtaccacatttgtagaggttttacttgctttaaaaaacctcccacacctccccctgaacctgaaacataaaatgaatgcaattgttgtt
gttaacttgtttattgcagcttataatggttacaaataaagcaatagcatcacaaatttcacaaataaagcatttttttcactgcattctagttgtggtttgtccaaac
tcatcaatgtatcttatcatgtctgctcgaagcggccggccatcaccactttgtacaagaaagctgggtataacttcgtatagcatacattatacgaagttatacta
gtgctatcgatgttgagctcaggtacctataacttcgtataggatactttatacgaagttattctagaatggctcgtactaagcagaccgctcggaaagccacagg
aggaaaggccccacggaaacaactggctacaaaagccgcccgcaagagcgccccggccaccggcggcgtgaagaagcctcaccgctaccgtcccggcaccg
tggcgctgcgcgagatccggcgctaccagaagtcgaccgagctgctgatccgcaagctgccgttccagcgcctggtgcgcgagatcgcgcaggacttcaagacc
gacctgcgcttccagagctcggccgtcatggctctgcaggaggcctgtgaggcctacttggttggcctcttcgaagataccaacctgtgcgccatccacgccaagc
gtgtcaccatcatgcccaaggacatccaactggcccgccgcatccgcggggagagggcgtaacctaggataacttcgtataatgtatgctatacgaagttatcaa
gcttgcgtacgcgtgaccggtactcgaggataacttcgtataaagtatcctatacgaagttatagcctgctttttgtacaaacttgccaacctgaaaaaaagtga

tttcaggcaggtgctccaggtaattaaacattaataccccaccaaccaaccatcccttaaacccttacctcttgctcagctaattacagcccggaggagaagggc

cgtcccgcccgctcacctgtgggagtaacgcggtcagtcagagccggggcgggcggcgcgaggcggcggcggagcggggcacggggcgaaggcagcgcgca

gcgactcccgcccgccgcgcgcttcgctttttatagggccgccgccgccgccgcctcgccataaaaggaaactttcggagcgcgccgctctgattggctgccgcc

gcacctctccgcctcgccccgccccgcccctcgccccgccccgccccgcctggcgcgccccccccccccccgcccccatcgctgcacaaaataattaaaaa

ataaataaatacaaaattgggggtggggaggggggggagatggggagagtgaagcagaacgtggggctcacctcgaccatggtaatagcgatgactaatacg

tagatgtactgccaagtaggaaagtcccataaggtcatgtactgggcacaatgccaggcgggccatttaccgtcattgacgtcaatagggggcgtacttggcata

tgatacacttgatgtactgccaagtgggcagtttaccgtaaatactccacccattgacgtcaatggaaagtccctattggcgttactattgacgtcaatgggcggg

ggtcgttgggcggtcagccaggcgggccatttaccgtaagttatgtaacgcaacttttctatacaaagttggagggcctatttcccatgattccttcatatttgcata

tacgatacaaggctgttagagagataattggaattaatttgactgtaaacacaaagatattagtacaaaatacgtgacgtagaaagtaataatttcttgggtagt

ttgcagttttaaaattatgttttaaaatggactatcatatgcttaccgtaacttgaaagtatttcgatttcttggctttatatatcttgtggaaaggacgaaacaccgT

GTCCTCAAACAGACCCACAgttttagagctagaaatagcaagttaaaataaggctagtccgttatcaacttgaaaaagtggcaccgagtcggtgcttttt

tggccggccgagggcctatttcccatgattccttcatatttgcatatacgatacaaggctgttagagagataattggaattaatttgactgtaaacacaaagatatt

agtacaaaatacgtgacgtagaaagtaataatttcttgggtagtttgcagttttaaaattatgttttaaaatggactatcatatgcttaccgtaacttgaaagtattt

cgatttcttggctttatatatcttgtggaaaggacgaaacaccgGCCGCCGGTGGACTTGCGAGgttttagagctagaaatagcaagttaaaataaggc

tagtccgttatcaacttgaaaaagtggcaccgagtcggtgcttttttggccggccgagggcctatttcccatgattccttcatatttgcatatacgatacaaggctgt

tagagagataattggaattaatttgactgtaaacacaaagatattagtacaaaatacgtgacgtagaaagtaataatttcttgggtagtttgcagttttaaaatta

tgttttaaaatggactatcatatgcttaccgtaacttgaaagtatttcgatttcttggctttatatatcttgtggaaaggacgaaacaccgGGTGGCTAGCTGC

TTGCGCGgttttagagctagaaatagcaagttaaaataaggctagtccgttatcaacttgaaaaagtggcaccgagtcggtgc
